# Treatment outcomes of acute streptococcal tonsillitis according to antibiotic treatment. A retrospective analysis of 242,366 cases treated in the community

**DOI:** 10.1080/13814788.2022.2083105

**Published:** 2022-06-13

**Authors:** Mattan Bar-Yishay, Ilan Yehoshua, Avital Bilitzky, Yan Press

**Affiliations:** a Department of Family Medicine, Maccabi Healthcare Services, Tel Aviv, Israel; b Department of Family Medicine and Siaal Research Center for Family Medicine and Primary Care, The Haim Doron Division of Community Health, Faculty of Health Sciences, Ben-Gurion University of the Negev, Beer-Sheva, Israel; c Maccabitech Institute of Research and Innovation, Maccabi Healthcare Services, Tel Aviv, Israel; d Department of Geriatrics, Soroka University Medical Center, Beer-Sheva, Israel

**Keywords:** Tonsillitis, pharyngitis, antibiotic treatment, upper respiratory tract infections, primary care

## Abstract

**Background:**

Acute upper respiratory infections are the most common reason for primary physician visits in the community. This study investigated whether the type of antibiotic used to treat streptococcal tonsillitis can reduce the burden by affecting the number of additional visits.

**Objectives:**

To assess the effect of different antibiotic treatments for tonsillitis on the number of additional primary physician visits and the development of infectious or inflammatory sequels.

**Methods:**

This retrospective study included first cases of culture-confirmed streptococcal tonsillitis (*n* = 242,366, 55.3% females, 57.6% aged 3–15 years) treated in primary clinics throughout Israel between the years 2010 and 2019. Primary outcomes were the number of additional primary physician visits, due to any cause or due to specific upper airway infections. Secondary outcomes were the number of developed complications, such as peritonsillar abscess, post-streptococcal glomerulonephritis, rheumatic fever, post-streptococcal arthritis, chorea and death.

**Results:**

Compared to penicillin-V, adjusted incidence rate ratios (IRR) for additional primary physician visits at 30–days were highest for IM benzathine-benzylpenicillin (IRR = 1.46, CI 1.33–1.60, *p* < .001) and cephalosporin treatment (IRR = 1.27, CI 1.24–1.30, *p* < .001). Similar results were noted for visits due to specific diagnoses such as recurrent tonsillitis, otitis media and unspecified upper respiratory tract infection. Amoxicillin showed decreased adjusted odds ratio (aOR) of developing complications (aOR = 0.68, CI 0.52–0.89, *p* < .01 for any complication. aOR = 0.75, CI 0.55–1.02, *p* = .07 for peritonsillar or retropharyngeal abscess).

**Conclusion:**

Penicillin-V treatment is associated with fewer additional primary physician visits compared to other antibiotic treatments. Amoxicillin and penicillin-V are associated with fewer complications. These findings are limited by the retrospective nature of the study and lack of adjustment for illness severity. Further prospective studies may be warranted to validate results.

KEY MESSAGES

- Treatment of acute streptococcal tonsillitis in the community with penicillin-V is associated with fewer additional physician visits than other antibiotics, due to infectious sequels or any cause.
- Treatment with amoxicillin or penicillin-V is associated with fewer complications.

## Introduction

Upper respiratory infections are the most common reason for urgent primary care physician visits [1,2]. While the majority of upper respiratory tract infections are of viral aetiology [3], up to 40% of annual antibiotic prescriptions in the community setting are due to acute respiratory infections [4], and more than 60% of adults present with a sore throat receive a prescription for antibiotic treatment [5,6]. Multiple treatment guidelines single out pharyngitis caused by Group A Beta-Haemolytic Streptococcus (GABHS) infection as an indication for antibiotic treatment [7,8], due to a reduction in both suppurative and non-suppurative sequels of GABHS pharyngitis [5,9,10]. European and Australian guidelines, however, recommend against routinely testing for GABHS or prescribing antibiotics in cases of sore throat due to the unlikely event of complications even if antibiotics are withheld [11–13].

Various studies have shown similar efficacy of amoxicillin and penicillin-V in successfully treating GABHS pharyngitis [14–16]. Since then, once-daily amoxicillin has been prescribed often [6], especially among children, presumably due to ease of administration and preferably tasting formulation. By comparison, IM benzathine penicillin is administered far less frequently. A single IM injection of benzathine penicillin is a long-acting treatment, with detectable levels of penicillin found in serum and tonsils for up to four weeks following injection [17]. Interestingly, benzathine penicillin is the only antibiotic therapy that has been shown to prevent acute rheumatic fever in controlled studies [18–20]; however, these were conducted in the 1950s. The few studies that have compared IM benzathine penicillin treatment to oral amoxicillin have reported superior GABHS eradication rates among children in low-resource countries [21,22], presumably due to increased compliance. Additional studies comparing benzathine penicillin to other antibiotics demonstrated similar clinical and biological cure rates between treatment groups [23,24]. However, despite efficiency, wide availability and low cost, benzathine penicillin is rarely utilised in the primary treatment of acute GABHS pharyngitis in high-resource community settings. The discomforts to patient, physician and parent associated with an IM injection are assumed to be the cause of low utilisation in high-resource countries, where compliance and rheumatic sequels are of less concern.

This study investigated whether the type of antibiotic used to treat GABHS tonsillitis affects the number of additional primary physician visits or the development of infectious or inflammatory sequels. Our initial hypothesis was that treatment with IM benzathine penicillin, due to its long-acting nature, would result in fewer additional visits due to infectious upper respiratory sequels.

## Methods

### Study design

This retrospective study included cases of GABHS tonsillitis who received appropriate antibiotic treatment in the community between 2010 and 2019, and investigated whether the type of antibiotic treatment used affected the number of additional physician visits, and the incidence of suppurative or non-suppurative sequels.

### Setting

Multi-center, including all primary care clinics of Maccabi healthcare services in Israel.

### Participants and data acquisition

Cases of GABHS tonsillitis treated in the community between the years 2010 and 2019 were identified using MD Clone system, which allows for case identification around a reference event. Initially, 420,954 patients given a clinical diagnosis of tonsillitis or pharyngitis in the community by a primary physician (family physician or paediatrician) were identified. Diagnoses included in our initial search are detailed in the Supplementary Materials (Appendix 1). Only the first case of clinically diagnosed pharyngitis for each patient identified within the 10-year study period was included. Cases in which the first diagnosis was recurrent tonsillitis/pharyngitis were excluded. Of the 420,954 clinically diagnosed cases identified, 283,092 (67%) patients had a positive throat culture result for GABHS within four days of clinical diagnosis. Out of those culture-confirmed GABHS tonsillitis cases, 242,366 (86%) patients purchased a suitable course of systemic antibiotic treatment within seven days of clinical diagnosis. Antibiotic treatments included are detailed in the Supplementary Materials (Appendix 2).

### Main outcomes and measures

Pre-defined primary outcomes were the number of additional primary physician visits within 30, 60 and 90 days from diagnosis, due to any cause or specific upper airway infections. Pre-defined secondary outcomes were the number of complications occurring within 90 days, such as peritonsillar or retropharyngeal abscess, post-streptococcal glomerulonephritis, rheumatic fever, acute rheumatic heart disease, post-streptococcal arthritis, chorea or death. Diagnoses included in or search for complications are detailed in the Supplementary Materials (Appendix 3).

### Statistical analysis

Statistical analysis was performed using IBM SPSS v27. There was no missing data for any variable among the 242,366 cases included in the analysis. Data were reported as mean and standard error of mean (SEM) for continuous variables and percentages or frequencies for categorical variables.

Chi-square test was used to examine the univariate effect of type of antibiotic on the number of physician visits and the number of complications recorded.

The adjusted effect of the type of antibiotic treatment on the number of physician visits was estimated using multivariate Poisson regression models and presented as incident rate ratios. Models were adjusted for age, sex, birth country, number of complications and prescribing physician specialty. No adjustment was possible for illness severity or physician propensity to prescribe.

The adjusted effect of the type of antibiotic treatment on the recorded occurrence of any complication, or the development of peritonsillar or retropharyngeal abscess, was estimated using multivariate Logistic regression models and presented as adjusted odds ratios. Models were adjusted for age, sex, birth country and prescribing physician speciality but not for illness severity or physician propensity to prescribe.

All *p* values were two-sided and statistical significance was set at *p* ≤ .05.

### Ethics

The study was approved by Maccabi Healthcare Services’ Helsinki committee.

## Results

Overall, 242,366 patients treated in the community for acute GABHS tonsillitis were identified and included in this analysis. All cases included had a positive throat culture for GABHS, were clinically diagnosed by a primary physician and were allocated a suitable course of systemic antibiotic treatment. Patient characteristics, antibiotics dispensed and the frequencies of complications at 90 days after diagnosis are summarised in Table 1.

**Table 1.** Patient characteristics, antibiotic treatment dispensed and rate of complications at 90 days follow-up.

Characteristics	%, (*n*)
Gender	** **
Male, % (*n*)	44.7% (108,327)
Female, % (*n*)	55.3% (134,039)
Age	
<3 years, % (*n*)	8.2% (19,880)
3–15 years, % (*n*)	57.6% (139,655)
16–45 years, % (*n*)	29.5% (71,527)
>46 years, % (*n*)	4.7% (11,304)
Antibiotic treatment	
Amoxicillin, % (*n*)	55.4% (134,266)
Phenoxymethylpenicillin, % (*n*)	32.6% (79,109)
Amoxicillin/clavulanate, % (*n*)	3.7% (9077)
Benzathine benzylpenicillin, % (*n*)	0.1% (267)
Cephalosporin, % (*n*)	1.9% (4601)
Macrolide, % (*n*)	6.2% (15,046)
Prescribing physician	
Primary	83% (202,120)
-Family physician	29% (70,063)
-Pediatrician	54% (132,057)
Secondary	0.37% (900)
-ENT	0.27% (669)
-Other secondary	0.1% (231)
Unknown	16% (39,346)
Complication rate, 90 days	
Peritonsillar and retropharyngeal abscess, % (*n*)	0.2% (379)
Post-streptococcal glomerulonephritis, % (*n*)	0.01% (21)
Rheumatic fever, % (*n*)	0.02% (46)
Acute rheumatic heart disease, % (*n*)	0.004% (10)
Post-streptococcal arthritis, % (*n*)	0.005% (13)
Chorea, % (*n*)	0.001% (3)
Death, any cause, % (*n*)	0.002% (4)

Most cases involved females (55.3%) and children aged 3–15 years old (57.6%) and the majority of cases were treated with amoxicillin (*n* = 134,266, 55.4%), followed by penicillin-V (*n* = 79,109, 32.6%). Children aged 3–15 (*n* = 139,655) were most likely to be treated with Amoxicillin (71.9%). By comparison, penicillin-V was dispensed in only 19.6% of cases involving children 3–15 years of age. Among adults aged 16–45 (*n* = 71,527), penicillin-V was most likely to be dispensed (62.4%), followed by amoxicillin (20.6%).

As expected, the overall number of complications recorded was low (Table 1). Of significance were the recorded 379 cases (0.16%) of peritonsillar or retropharyngeal abscess and the 46 cases (0.02%) of rheumatic fever.

### Additional primary physician visits according to antibiotic treatment

A multivariate analysis was performed adjusting for age, sex, country of birth, complications and prescribing physician specialty. No adjustment was possible for illness severity or physician propensity to prescribe. Univariate analysis is detailed in Supplementary Materials (Appendix 4). Results of multivariate analysis are summarised in Table 2. Adjusted incidence rate ratios (IRR) for additional primary physician visits due to any cause were highest for patients treated with IM benzathine benzylpenicillin (30 day IRR = 1.46, CI 1.33–1.60, *p* < .001) or a cephalosporin (30 day IRR = 1.27, CI 1.24–1.30, *p* < .001). Amoxicillin treatment showed similar results to penicillin-V (30 day IRR = 1.07, CI 1.06–1.08, *p* < .001). Penicillin-V treated patients also showed a decreased number of follow-up visits due to specific upper airway diagnoses, such as sore throat, tonsillitis/pharyngitis, otitis media and unspecified upper respiratory tract infection (Table 2).

**Table 2.** Multivariate analysis of additional visits according to antibiotic treatment.

	Antibiotic treatment	Adjusted OR (95%CI)	PV
Additional visits, any cause, at 30 days	Phenoxymethylpenicillin	Reference	
	Amoxicillin	1.07 (1.06–1.08)	<0.001
	Amoxicillin/clavulanate	1.12 (1.09–1.14)	<0.001
	Benzathine benzylpenicillin	1.46 (1.33–1.60)	<0.001
	Cephalosporin	1.27 (1.24–1.30)	<0.001
	Macrolide	1.15 (1.13–1.17)	<0.001
Additional visits, any cause, at 60 days	Phenoxymethylpenicillin	Reference	
	Amoxicillin	1.09 (1.08–1.10)	<0.001
	Amoxicillin/clavulanate	1.13 (1.11–1.15)	<0.001
	Benzathine benzylpenicillin	1.39 (1.28–1.51)	<0.001
	Cephalosporin	1.27 (1.24–1.30)	<0.001
	Macrolide	1.15 (1.13–1.16)	<0.001
Additional visits, any cause, at 90 days	Phenoxymethylpenicillin	Reference	
	Amoxicillin	1.10 (1.09–1.11)	<0.001
	Amoxicillin/clavulanate	1.12 (1.11–1.14)	<0.001
	Benzathine benzylpenicillin	1.36 (1.27–1.46)	<0.001
	Cephalosporin	1.26 (1.23–1.28)	<0.001
	Macrolide	1.16 (1.15–1.17)	<0.001
Additional visits, due to pharyngitis, tonsillitis or sore throat, at 90 days	Phenoxymethylpenicillin	Reference	
	Amoxicillin	1.02 (1.01–1.03)	<0.001
	Amoxicillin/clavulanate	1.18 (1.15–1.20)	<0.001
	Benzathine benzylpenicillin	1.55 (1.40–1.71)	<0.001
	Cephalosporin	1.18 (1.15–1.21)	<0.001
	Macrolide	1.12 (1.10–1.14)	<0.001
Additional visits due to otitis media, at 90 days	Phenoxymethylpenicillin	Reference	
	Amoxicillin	1.59 (1.5–1.68)	<0.001
	Amoxicillin/clavulanate	2.38 (2.14–2.64)	<0.001
	Benzathine benzylpenicillin	0.67 (0.30–1.50)	0.33
	Cephalosporin	1.85 (1.61–2.12)	<0.001
	Macrolide	1.76 (1.62–1.91)	<0.001
Additional visits due to upper respiratory tract infection, at 90 days	Phenoxymethylpenicillin	Reference	
	Amoxicillin	1.20 (1.17–1.22)	<0.001
	Amoxicillin/clavulanate	1.17 (1.11–1.22)	<0.001
	Benzathine benzylpenicillin	1.23 (0.98–1.53)	0.067
	Cephalosporin	1.17 (1.10–1.24)	<0.001
	Macrolide	1.19 (1.15–1.23)	<0.001
Additional visits due to acute sinusitis, at 90 days	Phenoxymethylpenicillin	Reference	
	Amoxicillin	0.98 (0.90–1.07)	0.67
	Amoxicillin/clavulanate	1.74 (1.55–1.95)	<0.001
	Benzathine benzylpenicillin	0.65 (0.21–2.03)	0.46
	Cephalosporin	1.31 (1.10–1.58)	0.003
	Macrolide	1.14 (1.00–1.31)	0.05

When directly comparing any oral penicillin treatment (penicillin-V, amoxicillin or amoxicillin with clavulanate, *n* = 222,452) to IM benzathine benzylpenicillin treatment (*n* = 267), the number of additional primary physician visits due to any cause was significantly lower with oral penicillin treatment. On multivariate analysis, oral penicillin-based treatment showed aIRR ratios of 0.72 (CI 0.65–0.79, *p* < .001) for additional visits at 30 days, IRR = 0.76 (CI 0.70–0.82, *p* < .001) at 60 days, IRR = 0.78 (CI 0.73–0.84, *p* < .001) at 90 days and IRR = 0.66 (CI 0.60–0.73, *p* < .001) for additional visits due to sore throat, tonsillitis or pharyngitis. The number of additional visits due to otitis media, unspecified upper respiratory tract infection or acute sinusitis was not significantly different on univariate or multivariate analysis.

When comparing any penicillin-based treatment (penicillin-V, amoxicillin, amoxicillin with clavulanate or IM benzathine benzylpenicillin, *n* = 222,719) to non-penicillin-based treatments (cephalosporin or macrolide, *n* = 19,647), non-penicillin treatment was associated with additional primary physician visits due to any cause, with an adjusted incidence rate ratio of 1.12 (CI 1.10–1.13, *p* < .001) at 30 days, IRR = 1.10 (CI 1.09–1.12, *p* < .001) at 60 days and IRR = 1.11 (CI 1.10–1.12, *p* < .001) at 90 days following diagnosis. For additional visits due to sore throat, tonsillitis or pharyngitis, non-penicillin-based treatment showed an adjusted IRR of 1.11 (1.10–1.13, *p* < .001). The number of additional visits due to otitis media, unspecified upper respiratory tract infection or acute sinusitis was not significantly different.

### Complications according to antibiotic treatment

The frequencies of recorded complications according to antibiotic treatment can be found in the Supplementary Material (Appendix 5). Only cases of peritonsillar or retropharyngeal abscess (*n* = 379) were of sufficient number to perform a separate analysis. An additional analysis was performed for any recorded complication (*n* = 465). Results of multivariate analysis are summarised in Table 3.

**Table 3.** Multivariate analysis of complication according to antibiotic treatment.

	Antibiotic treatment	Adjusted OR (95%CI)	PV
Peritonsillar or retropharyngeal abscess	Phenoxymethylpenicillin	Reference	
	Amoxicillin	0.75 (0.55–1.02)	0.07
	Amoxicillin/clavulanate	6.26 (4.85–8.09)	<0.001
	Benzathine benzylpenicillin	8.61 (2.71–27.38)	<0.001
	Cephalosporin	2.34 (1.43–3.84)	0.001
	Macrolide	1.85 (1.27–2.69)	0.001
Any complication	Phenoxymethylpenicillin	Reference	
	Amoxicillin	0.68 (0.52–0.89)	<0.01
	Amoxicillin/clavulanate	5.28 (4.15–6.71)	<0.001
	Benzathine benzylpenicillin	10.77 (4.37–26.56)	<0.001
	Cephalosporin	2.75 (1.82–4.13)	<0.001
	Macrolide	1.41 (0.98–2.02)	0.06

Compared to penicillin-V, benzathine benzylpenicillin treated patients had the highest odds of developing a peritonsillar or retropharyngeal abscess, with an adjusted odds ratio (aOR) of 8.61 (CI 2.71–27.38, *p* < .001). For the development of any complication, benzathine benzylpenicillin treated patients showed an adjusted odds ratio of 10.77 (CI 4.37–26.56, *p* < .001) compared to penicillin-V treatment. Patients treated with amoxicillin with clavulanate also recorded significantly more complications, with aOR of 6.26 for peritonsillar or retropharyngeal abscess (CI 4.85–8.09, *p* < .001) and 5.28 for any complication (CI 4.15–6.71, *p* < .001), compared to penicillin-V.

Interestingly, patients treated with amoxicillin showed decreased odds of developing complications even compared to penicillin-V. Amoxicillin showed an aOR of 0.68 (CI 0.52–0.89, *p* < .01) for any complication and an aOR of 0.75 (CI 0.55–1.02, *p* = .07) for peritonsillar or retropharyngeal abscess, compared to penicillin-V. However, the latter was found statistically insignificant. Significantly increased odds were also noted for complications with cephalosporin or macrolide treatment (Table 3).

When directly comparing any oral penicillin treatment (penicillin-V, amoxicillin or amoxicillin with clavulanate) to IM benzathine benzylpenicillin treatment, the number of complications was significantly greater with benzathine benzylpenicillin treatment, showing an aOR of 8.97 (CI 3.64–22.08, *p* < .001) for any complication and 6.30 (CI 1.98–20.04, *p* = .002) for peritonsillar or retropharyngeal abscess.

When comparing any penicillin-based treatment (penicillin-V, amoxicillin, amoxicillin with clavulanate or IM benzathine benzylpenicillin), to non-penicillin treatments (cephalosporin or macrolide), number of complications was significantly greater with non-penicillin-based treatments, with an aOR of 1.51 (CI 1.16 − 1.98, *p* = .002) for any complication and aOR = 1.53 (CI 1.14–2.05, *p* = .005) for peritonsillar or retropharyngeal abscess.

## Discussion

### Main findings

Our analysis showed fewer additional primary physician visits and complications associated with penicillin-V treatment compared to other antibiotics. A reduced number of complications was seen with amoxicillin use, even compared to penicillin-V. Penicillins in general were associated with better outcomes than macrolide or cephalosporin treatment. IM benzathine benzylpenicillin treatment was associated with increased additional primary physician visits and the greatest number of complications, followed by amoxicillin with clavulanate.

### Comparison with existing literature

In line with our findings of increased rates of re-consultations and complications associated with non-penicillin-based treatments, increased relapse rates of pharyngitis have been reported in some previous studies with both macrolide and cephalosporin treatments [25,26]. However, most previous studies reported similar clinical relapse rates and similar bacteriological cure rates with these treatments [10,27–29]. An explanation for our results may be the rising prevalence of GABHS strains resistant to macrolides and cephalosporins, which have been reported as an increasing cause of treatment failure [30–32].

Regarding our findings of increased re-consultations and complications associated with IM benzathine benzylpenicillin and amoxicillin with clavulanate, it was noted in our cohort that antibiotics other than penicillin-V and amoxicillin were more frequently prescribed by ENT specialists and that these cases were associated with more complications. It is very likely that initial disease severity affected choice of treatment. No measure of illness severity was included in our analysis, and no adjustment was possible to account for this confounding factor. Individual physician characteristics, habits and environment likely affected the choice of treatment as well [33–36]. Unfortunately, we could not adjust our analysis according to individual physician prescribing habits. It should be noted that previous studies have shown similar clinical and bacteriological cure rates with benzathine penicillin or amoxicillin with clavulanate, compared to other antibiotics [21–23]. We have no hypothesis other than reversed causality to explain our results regarding benzathine penicillin and amoxicillin with clavulanate treatment.

Of special interest and possible clinical significance among our findings was the reduced number of peritonsillar abscesses and other complications seen with amoxicillin use, even compared to penicillin-V. This suggests that among young adults, who are most often treated with penicillin-V, but are more likely than children to develop complications, treatment with amoxicillin may be preferable to penicillin-V. However, amoxicillin showed higher re-consultation rates compared to penicillin-V in our study, and the potential for adverse effects with amoxicillin treatment in the case of infectious mononucleosis (i.e. acute glandular fever) should be taken into account. In line with our findings of reduced follow-up visits associated with penicillin-V, a study by Moore et al. showed that prescriptions other than penicillin-V, including amoxicillin, were associated with a greater risk of re-consultations within four weeks [37]. While the rate of complications seen in our study are similar to rates described in previous studies [5,38–40], studies comparing complication rates with different antibiotic treatments are scarce and underpowered [10], and non-suppurative sequels were not described at all in these studies. Our search could not identify any studies supporting or contradicting our findings of reduced complications with amoxicillin treatment.

### Strengths and limitations

Our study has several strengths, from the large number of cases in our analysis, to the completeness of the data in the electronic medical records. Furthermore, inclusion criteria resulted in a cohort of cases most representative of the acute pharyngitis cases commonly encountered by primary physicians in the community. It should be noted that treatment according to throat culture results is a common clinical practice in the setting of our study, while treatment is often based on clinical scores without routine swabbing in other countries. Studies inspecting outcomes of clinical tonsillitis, adjusted for clinical score and illness severity, may be more relevant in such populations. Antibiotic use in our study, however, closely resembled physician prescription habits described in previous international studies [6,41,42].

The study is significantly limited by its observational retrospective design, rendering any associations found without clear causality. Furthermore, no adjustments were made for illness severity or propensity to prescribe. Clinical score on presentation was not reported and no other measure of illness severity was accounted for; hence treatment choice could have been influenced by illness severity. No data regarding symptomatic recovery was available. Additionally, only the first antibiotic dispensed around the diagnosis of tonsillitis was accounted for, and any second-line treatment received by patients would have skewed results. Smoking status, a well-documented risk factor for upper respiratory infections, recurrent tonsillitis and peritonsillar abscess, was also not accounted for. Neither were other comorbidities which may have affected outcomes nor any antibiotic allergies which may have affected the treatment choice. Data regarding complications were obtained from the electronic medical records of Maccabi healthcare services, which do not automatically include diagnoses from hospital and emergency department visits, unless recorded by any community physician during a follow-up visit after discharge. This would have caused an underrepresentation of complications in the cohort. Importantly, benzathine penicillin treatment represented only a tiny portion of cases in our cohort; hence the significance of any findings regarding benzathine penicillin are in doubt.

## Conclusion

Treatment of acute GABHS tonsillitis in the community with oral penicillin-V is associated with fewer additional primary physician visits compared to other antibiotic treatments, both due to specific upper respiratory infections and due to any cause. Treatment with amoxicillin may be associated with reduced development of the rare but serious complications of GABHS tonsillitis compared to other antibiotic treatments, including penicillin-V. Overall, treatment with any penicillin-based antibiotic is associated with a reduced number of complications and a reduced number of additional primary physician visits, compared to non-penicillin-based treatment. However, results should be interpreted with care due to the limitations of this retrospective analysis of observational data, performed without adjusting for illness severity or propensity to prescribe. Additional prospective studies may be warranted to corroborate these results.

## Abbreviations

GABHS: Group A Beta-Hemolytic Streptococcal
IM: intramuscular
IRR: incidence rate ratio
aOR: adjusted odds ratio
